# Treatment of Multi-Focal Epilepsy With Resective Surgery Plus Responsive Neurostimulation (RNS): One Institution's Experience

**DOI:** 10.3389/fneur.2020.545074

**Published:** 2020-10-29

**Authors:** Diem Kieu Tran, Demi Chi Tran, Lilit Mnatsakayan, Jack Lin, Frank Hsu, Sumeet Vadera

**Affiliations:** ^1^Department of Neurological Surgery, University of California, Irvine, Irvine, CA, United States; ^2^Department of Neurology, University of California, Irvine, Irvine, CA, United States

**Keywords:** epilepsy, lobectomy, temporal, surgery, robotic, responsive neurostimulation (RNS)

## Abstract

**Objective:** Patients with medically refractory focal epilepsy can be difficult to treat surgically, especially if invasive monitoring reveals multiple ictal onset zones. Possible therapeutic options may include resection, neurostimulation, laser ablation, or a combination of these surgical modalities. To date, no study has examined outcomes associated with resection plus responsive neurostimulation (RNS, Neuropace, Inc., Mountain View, CA) implantation and we describe our initial experience in patients with multifocal epilepsy undergoing this combination therapy.

**Methods:** A total of 43 responsive neurostimulation (RNS) devices were implanted at UCI from 2015 to 2019. We retrospectively reviewed charts of patients from the same time period who underwent both resection and RNS implantation. Patients were required to have independent or multifocal onset, undergo resection and RNS implantation, and have a minimum of six-months for follow-up to be included in the study. Demographics, location of ictal onset, location of surgery, complications, and seizure outcome were collected.

**Results:** Ten patients met inclusion criteria for the study, and seven underwent both procedures in the same setting. The average age was 36. All patients had multifocal ictal onset on video electroencephalogram or invasive EEG with four patients undergoing subdural grid placement and four patients undergoing bilateral sEEG prior to the definitive surgery. Five patients underwent resection plus ipsilateral RNS placement and the remainder underwent resection with contralateral RNS placement. Two minor complications were encountered in this group. At six months follow up, there was an average of 81% ± 9 reduction in seizures, while four patients experienced complete seizure freedom at 1 year.

**Conclusion:** Patients with multifocal epilepsy can be treated with partial resection plus RNS. The complication rates are low with potential for worthwhile seizure reduction.

## Introduction

Approximately one-third of epilepsy patients have seizures that are refractory to antiepileptic medications (1, 2). Left untreated, patients are at risk of developing multiple comorbidities, and potentially death (3). For this reason, patients with medically refractory focal epilepsy should be referred to a Level 4 NAEC (National Association of Epilepsy Centers) center to be evaluated for surgical candidacy. The best outcomes are seen with temporal lobectomy for mesial temporal sclerosis (MTS) or other lesional resections, with 68% of patients with MTS becoming seizure free and 50% of neocortical extratemporal resections becoming seizure free at 2 years (4).

After being diagnosed with medically refractory epilepsy, pre-surgical evaluation includes video electroencephalography (EEG) monitoring to localize the ictal onset zone(s). If ictal onset is difficult to localize, or if bilateral or eloquent area ictal onset is suspected, the patients move on to have invasive monitoring studies including stereoelectroencephalography (sEEG) or subdural grids (SDG) to better delineate the ictal onset zone(s). Depending upon the location of the onset zone, subsequent resection, neurostimulation, or laser ablation might be performed.

However, there are limitations associated with performing resections including the inability to perform bilateral resection of the same lobe and the unfavorable functional outcomes associated with resecting eloquent regions. Therefore, patients with medically refractory epilepsy which involve multiple independent ictal onset zones or eloquent areas can be very difficult to treat surgically. Historically, when there is bilateral or multifocal ictal onset, palliative procedures such as callosotomy or neuromodulation have been performed (5–9). Unfortunately, these rarely result in complete seizure freedom. The authors describe their experience with resection of primary ictal onset zone as well as RNS implantation for additional ictal onset areas. This combination approach provides the ability to expand the treatment area outside of what is possible with resection alone. The authors demonstrate their early series of patients who underwent resection and RNS as part of their surgical management.

## Methods

### Patient Selection

A total of 43 responsive neurostimulation (RNS) were placed at UCI from 2013 to 2019. To be included in the study, patients must have independent multifocal ictal onset which was demonstrated with video encephalography (vEEG) or invasive monitoring (sEEG or SDG) and undergone resection plus RNS implantation. Resection and RNS implantation were not required to be performed during the same surgical setting. Subjects must also have at least six months of follow up. Between February 2015 to January 2019, 10 patients met inclusion criteria and underwent responsive neurostimulation implantation as well as a resective procedure. Eight out of ten patients underwent phase two invasive monitoring with either SDG placement and sEEG or bilateral sEEG, while two patients underwent definitive treatment without undergoing invasive monitoring (Tables 1, 2). This study was approved by the University of California, Irvine's Institutional Review Board (IRB).

**Table 1:** Patient characteristic.

**Pt No**	**Age at onset, Sex**	**No baseline seizures/month**	**Invasive EEG type**	**No of clinical seizures/month at 6 months follow up**	**No of clinical seizures/month at 1 year**	**No of clinical seizures/month at 2 years**
JA	1, M	Daily	SDG	8.2	7.8	0
VO	22, M	6	sEEG	0.16	0.08	0
RR	42, F	12	sEEG	2.6	6.8	3
JH	23, F	Daily	sEEG, then SDG^*^	7.5	7.1	1
MC	19, F	Daily	SDG with sEEG	6.8	6.1	1
JR	19, F	5	sEEG	1.2	1.2	
JW	8, F	Daily	sEEG, then SDG^*^	0.16	0.08	
EA	39, F	9	No invasive EEG done initially, then sEEG^**^	2.2	2.1	
SL	5, F	6	sEEG	1.2	0.08	
MM	0, F	8	No invasive EEG done	2.1	0.04	

* *Patients initially underwent bilateral sEEG, which lateralized to one side, then were taken back to surgery for SDG on the side that sEEG lateralized to*.

** *Patient initially underwent vEEG that showed left temporal ictal onset. Patient continued to have seizures after left anterior temporal lobectomy, so she was taken back for contralateral sEEG*.

**Table 2:** Clinical data.

**Pt**	**Semiology**	**MRI results**	**Neuropsychiatric testing**	**AEDs tried**	**Current AEDs**
JA	Begins with staring and unresponsiveness, progressing to pursing of his lips, followed by repetitive opening and closing hands	Left encephalomalacia in left insula/parietal region	Deficits in bilateral fine motor coordination, working memory, selective attention, visuomotor and verbal processing speed, confrontation naming, verbal fluency, visuospatial processing, contextual verbal learning and memory, visuomotor set-shifting, and complex reasoning	Lamotrigine, Levetiracetam, Topiramate XR, Lacosamide	Levetiracetam, Lamotrigine, Lacosamide
VO	Arousing from sleep or rest, shifting in bed, kicking his legs, picking and grabbing at his blanket, wiping face and nose with his right and at times left hand	Encepholomacia in b/l frontal lobes, corpus collosum, and left anterior temporal region	Diffuse cognitive impairment including impaired attention, processing speed, executive function, and expressive language abilities	Topiramate, Vimpat, Brivaracetam	Brivaracetam
RR	1. Aura, twitching of lips, which spread to right hand 2. Bilateral upper extremity tonic extension and posturing with repetitive movements	Mild bilateral MTS	Diffuse deficits across language, graphomotor, reasoning, processing, speed, attention, executive functioning, memory ability	Topiramate, Clonazapam, Lacosamide, Phenobarbital	Topiramate, Brivaracetam
JH	Started with strange behavior, confusion, followed by a febrile illness leading to a convulsive status epilepticus, now semiology is tingling, burning sensation in the right lower extremity ascending to the right upper extremity at times also face for a few second duration	Non lesional	Mild cognitive and memory impairment	Levetiracetam, Lacosamide, Clonazapam	Levetiracetam
MC	Confusion and speech difficulty followed by loss of awareness with secondary generalization	Left temporal cyst, which was resected, leaving empty cavity	Severe impairments in memory and learning, sustained and divided attention, mental flexibility, bilateral fine motor speed and dexterity, receptive and expression language skulls, and reading comprehension	Zonisamide, Lamotrigine, Lacosamide, Perampanel	Zonisamide, Lamotrigine
JR	Aura: sometimes deja vu 1. Spaced out, arms clenched, head to the left, drooling 2. Generalized tonic-clinic activity, tongue biting	Moderate left MTS, mild right MTS	Mild cognitive and memory impairment	Levetiracetam, Lamotrigine, Eslicarbazepine, Lacosamide	Levetriacetam
JW	Touching her bilateral temporal head regions, followed by being confused and turning her body to the left side along the horizontal body axis	Non lesional	Impaired verbal working memory and naming	Carbamazepine, Oxcarbazepine, Topiramate, Clonazapam, Acetazolamide	Lamotrigine, Levetiracetam
EA	Arrest in behavior, gaze preference to the left with repetitive hand movements, more commonly with the right	Non lesional	Impaired expressive vocabulary, visual memory, bilateral fine motor speed, and verbal reasoning	Carbamazepine, Valproic Acid, felbamate, Lamotrigine, Phenyltoin, Topiramate, phenobarbital, Oxcarbamazepine, Lacosimide, Zonisamide	Levetiracetam, Eslicarbazepine
SL	Loud laughter followed by screaming, then progressed to rocking body back and forth	Right frontal encephalomalacia with ventricular dilation (patient had prior right frontal lobectomy from a different institution with persistent seizures)	Impaired fine motor speed and dexterity, working memory and verbal learning	Lamotrigine, Perampanel	Lamotrigine
MM	Aura of fear, anxiety, and impending doom, staring, unresponsiveness and seen walking around and repeating words	Left MTS	Impairments in mental flexibility, problem-solving, phonemic, fluency, and divided attention. Intact visual and recognition memory	Carbamazepine, Topiramate, Brivaracetam, Lacosimide	Lacosimide, Carbazmazepine

### Presurgical Workup

All patients completed neuroimaging, neuropsychological testing, and non-invasive vEEG as part of their phase one evaluation. They were then presented and discussed at our institution's multidisciplinary epilepsy management conference. Epileptologists, neuroradiologists, and neuropsychologists as well as the senior author (SV) were present during these conferences. The subjects were presented by their treating epileptologist, and all relevant imaging studies, vEEG clips, and neuropsychological tests were thoroughly discussed. A treatment consensus was reached amongst all members of the group. After invasive monitoring was complete, patients with complex seizure onset (i.e., bilateral, eloquent area, or multifocal ictal onset) were again discussed at epilepsy management conference and a treatment consensus was reached. Once treatment consensus was reached, the patient was scheduled for definitive surgery within two weeks. All patients in the study underwent resection plus RNS implantation, although these were not necessarily performed during the same surgery.

### Surgical Technique

Patients first undergo either sEEG or SDG implantation to localize the ictal onset. If sEEG is performed and it is determined that they will require resection plus RNS, the patients are discharged home and brought back at a later date for the definitive surgery. If SDG is performed, the definitive surgery is performed upon removal of electrodes. Resections were performed after stereotactic RNS implantation to avoid risk of brain shift intraoperatively.

**Figure 1:**
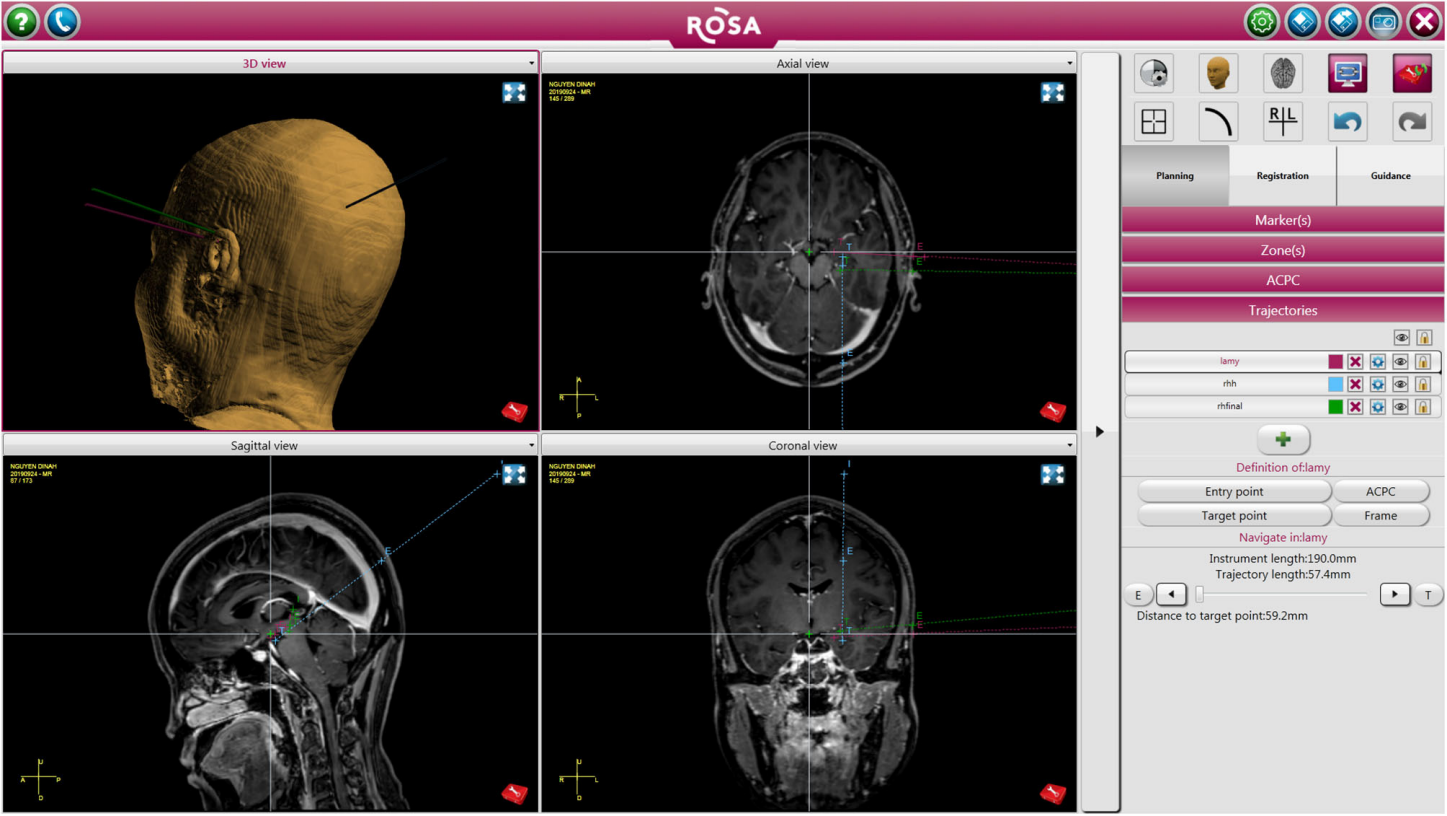
Patient undergoing left sided RNS placement with right temporal lobectomy. The figure depicts trajectory and target points for the RNS leads.

In patients that required depth electrode placement for RNS, stereotactic implantation of RNS was done using the Robotic Operating Surgical Assistant (ROSA) (Zimmer-Biomet, Warsaw, IN). A mayfield skull clamp was placed on the patient's head for immobilization. A curvilinear incision was marked in a location that was accessible to both leads and not interfere with reopening for future generator replacements, which served as the incision for placement of the RNS generator. The ictal onset zone identified by previously implanted sEEG electrodes were used to target RNS leads. All target and entry points were planned on the ROSA on the day of surgery (Figure 1).

The skull clamp was then attached to the robot with the patient in the correct position, and registration was performed using the robot's laser registration. Following registration, the robotic arm was used to identify the two entry points. A hand drill was utilized to access the cranial vault, and the dura was carefully opened with a dural probe. The robotic arm maintained a rigid trajectory to the target while an outer cannula and subsequently the RNS lead were passed. Each lead was secured in place using a dog bone-shaped plate from a cranial plating system, which has been previously described in a separate paper (10). The curvilinear incision was then opened, and a small craniectomy was created to hold the RNS generator. The device was then connected to the implanted leads.

For patients that required strip electrode placement for RNS, stereotactic implantation was done using the Stryker (Stryker Corporation, Kalamazoo, MI) neuro-navigation system. Again, a Mayfield skull clamp was applied and the images were uploaded onto the navigation system. Target areas were selected based on patient's SDG data. Patients requiring strip electrodes most commonly underwent temporal lobectomy and RNS placement on the ipsilateral side. The electrodes can be placed once the resection is completed and secured into place by anchoring it to the craniotomy site edges. The rest of the procedure is done in the similar way as described above.

**Figure 2:**
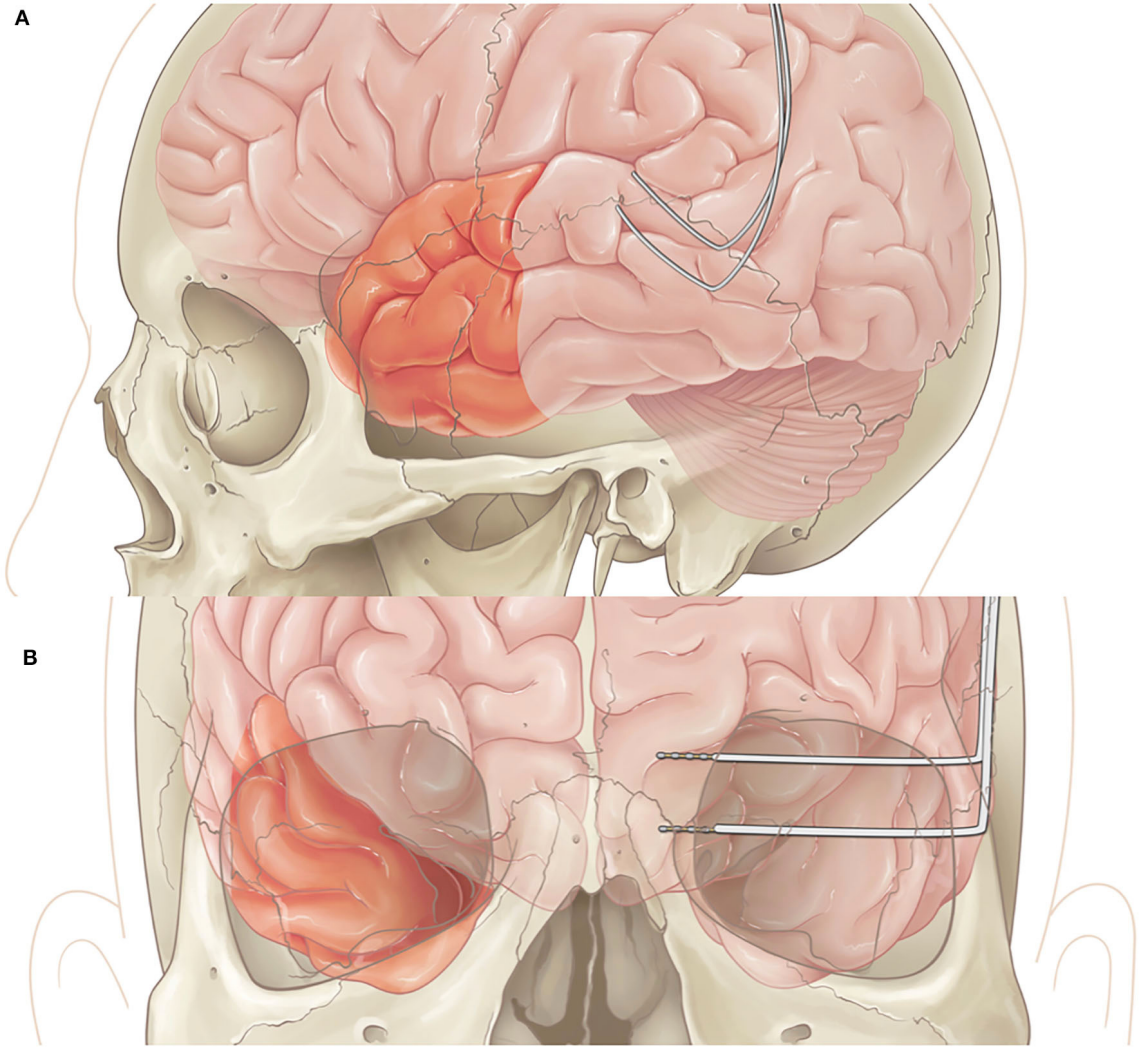
**(A)** Lateral view of left anterior temporal lobectomy with RNS placement in posterior temporal area. **(B)** Coronal view of right temporal lobectomy with RNS placement in left mesial structures.

In patients undergoing both resection and RNS placement, once the RNS is implanted successfully, the resection is performed based upon the area of interest with standard surgical techniques (Figures 2A,B).

An intraoperative CT is performed after the procedure and the image is fused with the planning MRI to confirm accurate placement of RNS leads.

### Data Collection

The clinical data of subjects who underwent resection plus RNS implantation from July 2015 to January 2019 was retrospectively collected and analyzed. Data for the following variables were collected: (1) age at seizure onset; (2) number of baseline seizures per month; (3) location of ictal onsets; (4) location of RNS leads and resection; (5) postoperative outcome; (6) ECoG from RNS; and (7) surgical complications.

## Results

The age range at the time of surgery was 27 to 53 years, with a mean age of 36 years (Table 3). There were two patients that underwent definitive treatment without first undergoing invasive monitoring because their vEEG showed clear independent bilateral ictal onset in one patient and left ictal onset in another patient. Four patients underwent SDG placement as part of the invasive monitoring, while the remainder underwent sEEG. Of the four patients that underwent SDG, two initially had sEEG that lateralized to one side and therefore patient returned for SDG implantation to better localize the onset zone. Five patients underwent RNS and ipsilateral resection and the remaining patients underwent contralateral RNS and resection. The patient with bilateral ictal onset later underwent wada testing which showed language dominance on the right, therefore she underwent left temporal lobectomy with right RNS placement. The patient that had only left ictal onset, underwent left temporal lobectomy but continued to have persistent seizures and therefore underwent bilateral sEEG, which showed right sided onset as well, therefore, she underwent right RNS placement. There were two minor postoperative complications; one patient developed an epidural hematoma under the RNS generator site requiring evacuation while the second had a cerebral spinal fluid leak that require lumbar drain placement (Table 2). No long-term sequela was noted and all patients returned to baseline. At six months of follow up, there was an average of 81% ± 9 decrease in seizure frequency, with two patients becoming seizure free. Four patients continued to remain seizure-free at their one year follow up with a total of 83% ± 17 seizure reduction in the study group. Two patients remained seizure free at 2 years follow up, while the rest have not had their 2 year follow ups yet. Seizure reduction was measured by subtracting the number of seizures per month at the time of follow up divided by the number of baseline seizures per month by the number one $\left\{\left[1-\left(\frac{\;number\;ofseizure\;per\;month\;at\;time\;of\;follow\;up}{number\;ofseizures\;per\;month\;at\;baseline}\right)\right]\times \;100\right\}$. Baseline seizures were calculated based on number of seizures patients had for 6 months.

**Table 3:** Surgical outcomes.

**Pt No**	**Location of ictal onset**	**Location of RNS leads and resection**	**ECoG from RNS**	**Complications**
JA	Left frontal with rapid spread to 3 left hippocampus	RNS strip electrodes in left frontal + left temporal lobectomy	Several areas of sharp waves, however, no clinical seizures seen	
VO	Right frontal, bilateral temporal	RNS depth electrodes in bilateral hippocampus + right frontal resection	Brief runs of sharp waves in right more than left temporal area, however, no clinical seizures	
RR	Bilateral temporal	RNS depth electrodes in right hippocampus + left temporal lobectomy	1-3 electroclinical seizures/month from right hippocampus, and runs of sharp waves seen in the same area	
JH	Left temporal	RNS strip electrodes in posterior left + left anterior temporal lobectomy	1-2 electroclinical seizures/month	
MC	Left temporal	RNS strip electrodes in posterior left + left anterior temporal lobectomy	3-5 electroclinical seizures/month, occasional prolonged runs of sharp waves	
JR	Left temporal, then right temporal	RNS depth electrodes in right hippocampus + left temporal lobectomy^β^	1-2 electrographic seizures from right hippocampus, runs of sharp waves from same area	
JW	Right frontal	RNS strip electrodes in anterior and posterior interhemispheric area + right frontal resection	1-2 brief electrographic seizures, but no clinical seizures	
EA	Left temporal, then right temporal	Right RNS depth electrodes in hippocampus + left temporal lobectomy^δ^	5-10 electrographic seizures from right hippocampus, and frequent runs of sharp waves	
SL	Right frontal	RNS strip electrodes in interhemispheric region near motor strip + right frontal resection	Several areas of sharp waves, no electroclinical seizure	CSF leak
MM	Bilateral temporal	RNS depth electrode into hippocampus + left temporal lobectomy^α^	Sharp waves from right hippocampus, no electroclinical seizures	EDH under RNS generator

α *Patient only underwent vEEG which showed bilateral independent ictal onset. She later underwent WADA testing which showed language dominance on right side, therefore she underwent left temporal lobectomy and right RNS placement*.

β *Patient initially underwent RNS implantation into bilateral hippocampus, however, patient continued to have frequent seizure. ECoG data of RNS shows persistent left sided seizures, therefore she underwent left temporal lobectomy*.

δ *Patient initially underwent left temporal lobectomy, however, patient continued to have frequent seizures. She then underwent contralateral sEEG, which showed independent ictal onset, therefore she underwent right RNS placement*.

*Patient had electrographic seizures on ECoG that correlated with patient's seizure diary or magnet swipe*.

## Discussion

Epilepsy surgery offers a potential cure for patients with medically refractory focal epilepsy (6, 9). Resections are considered when the seizure focus can be removed with minimal risk of disabling neurological deficits. Thus, patients with multifocal ictal onset zones, ictal onsets in eloquent areas, or bilateral ictal onsets still pose a significant challenge and these patients can be significantly more difficult to treat. The RNS device provides us with the ability to treat patients with multifocal and eloquent region onset (5). In this case series, we were able to supplement resective surgery with RNS in patients in which resective surgery alone could not address the entire ictal onset zone, such as in eloquent areas or with bilateral onset zones.

Patients with multifocal epilepsy historically were thought to be poor surgical candidates (11–13). There have been a few case series that show that patients with bilateral temporal lobe epilepsy may benefit after unilateral surgery (14–16), however, determining which side to resect also poses a challenge. In patients that have bilateral temporal lobe epilepsy, the chance of good seizure outcome after surgery (Engel class I or II) (17) is still much lower than patients with unilateral temporal lobe epilepsy at about 25–45% depending on the study (12, 18, 19). In our patients with bilateral ictal onset, we were able to use invasive EEG to lateralize which side demonstrated greater seizure activity. Because resection has been shown to have the best seizure free outcomes (6), we chose to treat the most active region with resection, while the contralateral side underwent RNS placement.

Patients with eloquent area onset (i.e., language or motor regions) pose an even greater challenge for treatment. In these patients, the risk of neurological deficit in resecting eloquent cortex outweigh the benefits of surgery. In these patients, partial resection plus RNS on the ipsilateral side can be done. This allows us to broaden the scope of coverage from the safe margin of the resection to eloquent regions with the RNS, effectively allowing us to extend our treatment of ictal onset outside of what is safe with resection alone.

**Figure 3:**
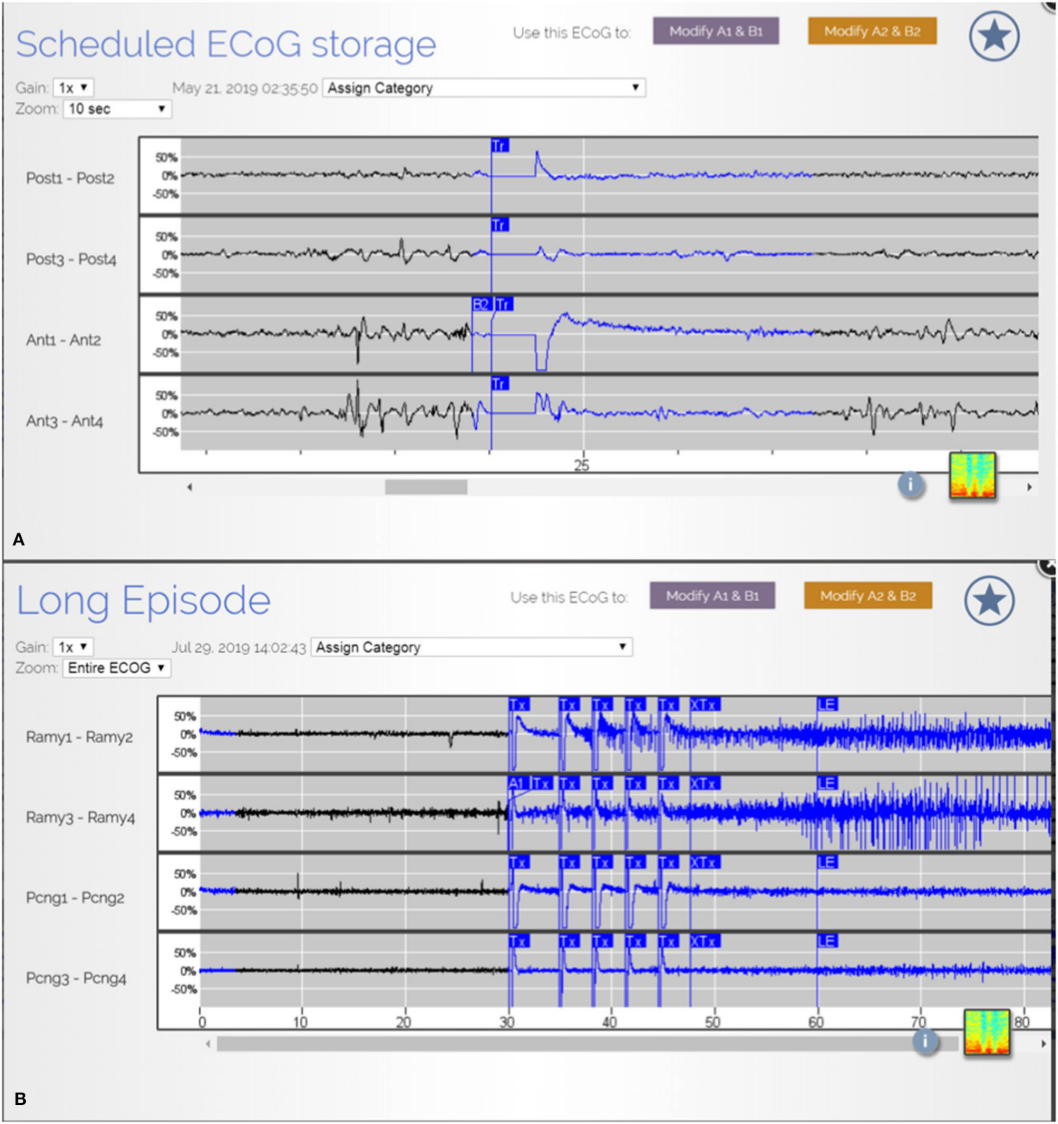
**(A)** ECoG from patient JA showing epileptiform discharges from multiple areas. **(B)** ECoG from patient R.R. showing detection and treatment of another electrographic seizure from right amygdala.

In our patient population, there was an overall reduction in clinical seizures post operatively. Despite this, at the most recent interrogation the RNS system recorded ongoing electrographic (Figures 3A,B) seizures in 60% of the patients and epileptiform discharges and patterns in all 10 patients from the remaining areas which were not able to be safely resected. Placement of the RNS system allowed for the detection and treatment of these residual activities. Partial resection of the seizure focus can help lessen the burden of ictal activities and reduce seizure frequency. However, when the complete seizure focus is not removed, there remains a propensity for seizure recurrences (6). The authors argue that supplementing resections with RNS to treat the residual epileptogenic regions can have a complementary effect and yield greater seizure reduction.

The results of this present study show that resection of ictal onset plus RNS implantation in another location is safe and efficacious. There were two minor complications that did not cause long-term deficits.

There are two main limitations of this study. One limitation is that the study group is not well powered with only 10 patients. Although our results provide valuable information regarding the safety and efficacy of resection plus RNS, the clinical outcomes data may not be statistically significant given the small sample. Additionally, there are only two patients included in the study who had long-term outcomes over one-year, and oftentimes, the clinical benefit from epilepsy surgery may take several years to fully manifest, especially in the setting of RNS placement. Nevertheless, neuromodulation often demonstrates improvements in seizure reduction over time, suggesting continued improvement in seizure control may be seen in this group in the future.

## Conclusion

RNS plus resection allows us to broaden the scope of coverage in patients with multifocal or eloquent ictal onset zones. While resection alone is limited by inability to perform bilateral resections or resections of eloquent regions, RNS provides the ability to extend the coverage and aid with seizure management in this difficult to treat patient population.

## Data Availability Statement

All datasets generated for this study are included in the article/Supplementary Material.

## Ethics Statement

The studies involving human participants were reviewed and approved by Cristobal Barrios, MD, vice chair of institutional review board at University of California Irvine. Written informed consent to participate in this study was not required according to local legislation and national guidelines.

## Author Contributions

DKT: developed the concept of the work, analysis of data, drafting the work, and critically revising the manuscript. DCT: developed the concept of the work, analysis of data, drafting the work, and critically revising the manuscript. LM, JL, and FH: revising the manuscript. SV: developed the concept of the work, analysis of data, drafting the work, critically revising the manuscript, and approved the manuscript for submission. All authors contributed to the article and approved the submitted version.

## Conflict of Interest

The authors declare that the research was conducted in the absence of any commercial or financial relationships that could be construed as a potential conflict of interest.
